# The Complexity of the Ovine and Caprine Keratin-Associated Protein Genes

**DOI:** 10.3390/ijms222312838

**Published:** 2021-11-27

**Authors:** Huitong Zhou, Hua Gong, Jiqing Wang, Yuzhu Luo, Shaobin Li, Jinzhong Tao, Jonathan G. H. Hickford

**Affiliations:** 1 International Wool Research Institute, Gansu Agricultural University, Lanzhou 730070, China; huitong.zhou@lincoln.ac.nz (H.Z.); luoyz@gsau.edu.cn (Y.L.); lisb@gsau.edu.cn (S.L.); 2 Gene-Marker Laboratory, Faculty of Agricultural and Life Sciences, Lincoln University, Lincoln 7647, New Zealand; gonghua3000@gmail.com; 3 Gansu Key Laboratory of Herbivorous Animal Biotechnology, College of Animal Science and Technology, Gansu Agricultural University, Lanzhou 730070, China; 4 Agriculture College, Ningxia University, Yinchuan 750021, China; tao_jz@nxu.edu.cn

**Keywords:** KAP, *KRTAP*, variation, polymorphism, wool, fibre, sheep, goat

## Abstract

Sheep (*Ovis aries*) and goats (*Capra hircus*) have, for more than a millennia, been a source of fibres for human use, be it for use in clothing and furnishings, for insulation, for decorative and ceremonial purposes, or for combinations thereof. While use of these natural fibres has in some respects been superseded by the use of synthetic and plant-based fibres, increased accounting for the carbon and water footprint of these fibres is creating a re-emergence of interest in fibres derived from sheep and goats. The keratin-associated proteins (KAPs) are structural components of wool and hair fibres, where they form a matrix that cross-links with the keratin intermediate filaments (KIFs), the other main structural component of the fibres. Since the first report of a complete KAP protein sequence in the late 1960s, considerable effort has been made to identify the KAP proteins and their genes in mammals, and to ascertain how these genes and proteins control fibre growth and characteristics. This effort is ongoing, with more and more being understood about the structure and function of the genes. This review consolidates that knowledge and suggests future directions for research to further our understanding.

## 1. Introduction

The keratin-associated proteins (KAPs) are structural components of the fibres that form the pelage of mammals. They are part of a matrix, which cross-links with the keratin (K) protein containing keratin intermediate filaments (KIFs), which are the other main structural components of the fibre. In the wool follicles of sheep, the KAPs are produced soon after the synthesis of keratins during the development of fibres in the follicle, and they are thought to cross-link with the KIFs by forming disulphide bonds with cysteine residues in the head and tail domains of the keratins [1]. The nature of this cross-linking is not well understood, and while co-immunoprecipitation studies have demonstrated an interaction between the head domain of human keratin K86 and KAP2-1 [2], and Western blot studies have demonstrated an interaction between the head domain of K85 and KAP8-1 [2], a complete understanding of which K and KAP cysteine thiol groups form disulphide bridges (be they inter- or intra-chain) is not well known, although the bulk of the most readily accessible cysteines in the KAPs are reported to be found close to either the N- or C-terminal domains in these proteins [3]. The KAPs are thought to play a critical role in regulating the physico-mechanical properties of the fibre.

With sheep and goats, the hair and wool fibres are produced by follicles that are located in the skin, but the value of these fibres varies considerably depending on their qualities, including their fineness (mean fibre diameter), their uniformity, their length, and their colour.

Since the first report of a complete KAP sequence in the late 1960s [4], considerable effort has been made to identify the KAP proteins and their genes in both sheep and goats. Research has also focused on ascertaining how these genes and proteins control and affect fibre qualities. Our understanding has progressed rapidly over the last two decades, especially since the sheep and goat genome sequences have become available. This has enabled more ovine and caprine KAP genes to be identified and characterized.

## 2. Keratin-Associated Proteins and the Genes That Encode Them

The KAPs are small (ca. 10–30 kDa) and possess either a high cysteine or a high glycine and tyrosine content [1,5]. These proteins were originally categorized into three broad groups based on their amino acid composition: the high sulphur (HS; ≤30 mol% cysteine), the ultra-high sulphur (UHS; >30 mol% cysteine) and the high glycine and tyrosine (HGT; 35–60 mol% glycine and tyrosine) KAPs [1]. Despite being glycine and tyrosine rich, the HGT-KAPs also contain an abundance of cysteine, with the apparent exception of KAP36-1, a recently identified protein from sheep that is deficient in cysteine [6].

The absence of cysteine in ovine KAP36-1 suggests the possibility of other forms of cross-linking or interaction for the HGT-KAPs. In this respect the presence of tyrosine in the KAPs may be of significance. Tyrosine is an aromatic amino acid and contains a benzene ring in its side-chain. The benzene ring may allow the tyrosine residues in the HGT-KAPs to interact with other tyrosine residues, or other aromatic amino acids in the KAPs and/or the KIFs, using a ‘ring-stacking’ mechanism. This cross-linking mechanism has been described for other aromatic amino acid-containing proteins [7]. In the HGT-KAPs, the tyrosine residues are usually surrounded by glycine residues. Having a small amino acid such as glycine in proximity to tyrosine would allow the tyrosine residues greater freedom to move their benzene rings into a preferred spatial orientation (i.e., conformational freedom), and thus enable the formation of amino acid to amino acid interactions. Tyrosine also possesses a reactive hydroxyl group at the end of its side-chain, which can act as a hydrogen donor, and thus potentially form hydrogen bonds with the centre of the benzene ring from another tyrosine, or another aromatic amino acid [8]. This would make the ring-stacking interaction even stronger, and may result in the wool fibre being further strengthened, while simultaneously giving some degree of pliability [9].

All of the KAPs are encoded by single exon (intron-less) genes called the *KRTAP*s and, accordingly, the *KRTAP*s are understandably small in size (with an open reading frame of usually less than 800 bp). They often share sequence similarities with each other, and can be assigned into families based on sequence similarity. Extensive bioinformatics analyses of the human genome have resulted in the identification of 88 functional *KRTAP*s, which is probably close to the complete catalogue of these genes in humans [10,11,12]. These *KRTAP*s have been assigned into 25 KAP families, numbered from KAP1 to KAP27, with the exclusion of KAP14 and KAP18. The KAP14 family name has been used for two murine HS-KAP genes, *KRTAP14-1* (previously named mKAP13) [13] and *KRTAP14-2* (previously called pmg1) [14], whereas the KAP18 family has been used for a different murine HGT-KAP gene [4]. Intact orthologues of these genes are not found in humans [15], suggesting that these *KRTAP*s may be mouse-specific.

**Figure 1:**
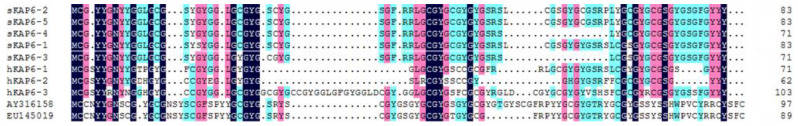
Comparison of the predicted amino acid sequences of two goat “*KRTAP6-2*” sequences with sheep and human *KRTAP6* sequences. The goat “*KRTAP6-2*” sequences are indicated with the GenBank accession number AY316158 and EU145019. The sheep sequences are indicated with the prefix “s”, while the human sequences are marked with the prefix “h”. The GenBank accession numbers of these sequences are NM_001193399 (sKAP6-1), KT725832 (sKAP6-2), KT725837 (sKAP6-3), KT725840 (sKAP6-4), KT725845 (sKAP6-5), NM_181602 (hKAP6-1), NM_181604 (hKAP6-2), and NM_181605 (hKAP6-3). The numbers on the right of the sequences represent the length of the proteins. Amino acid positions with high levels of homology are coloured, with black indicating 100% homology, red indicating greater than or equal to 75%, and blue indicating greater than or equal to 50%.

Numerous *KRTAP*s have been described in sheep and goats, with the identification of 30 ovine *KRTAP*s and 18 caprine *KRTAP*s to date. These *KRTAP*s represent 18 ovine KAP gene families (Table 1) and 12 caprine KAP families (Table 2). There are four other ovine *KRTAP* sequences reported that probably represent four other *KRTAP*s in sheep [16], but because these sequences are only described as partial coding sequences, their identity awaits further investigation. They are, accordingly, not included in the ‘identified’ *KRTAP*s described in Table 1. Two other goat *KRTAP* sequences, EU145019 [17] and AY316158 [18], are reported to be “alleles of caprine *KRTAP6-2*”, but a sequence analysis suggests that these two sequences are quite different to the *KRTAP6-n* sequences from sheep and humans (Figure 1). This suggests that these may not be caprine *KRTAP6-n* sequences, especially given other similarities between the sheep and goat genomes at the nucleotide sequence level, albeit not at the level of chromosomal arrangement. Accordingly, their true identity appears to await further investigation.

Orthologues for all of the human *KRTAP*s can be found in sheep and goats, with the exception of *KRTAP25-1*. A recent search for sequences comparable to human *KRTAP25-1* in the sheep genome assembly did not reveal any comparable sequences [47], but in the chromosome region, where the human *KRTAP25-1* orthologue was expected to be found, there was an apparently unique *KRTAP* sequence, which has been named *KRTAP28-1* [47].

In contrast, several *KRTAP*s that have not been described in humans are found in sheep and goats, including *KRTAP8-2* [35,55], *KRTAP6-4* [31], *KRTAP6-5* [31], and *KRTAP36-1* [6]. All of these ‘additional’ *KRTAP*s encode HGT-KAPs, which suggests that sheep possess more HGT-*KRTAP*s than humans.

**Figure 2:**
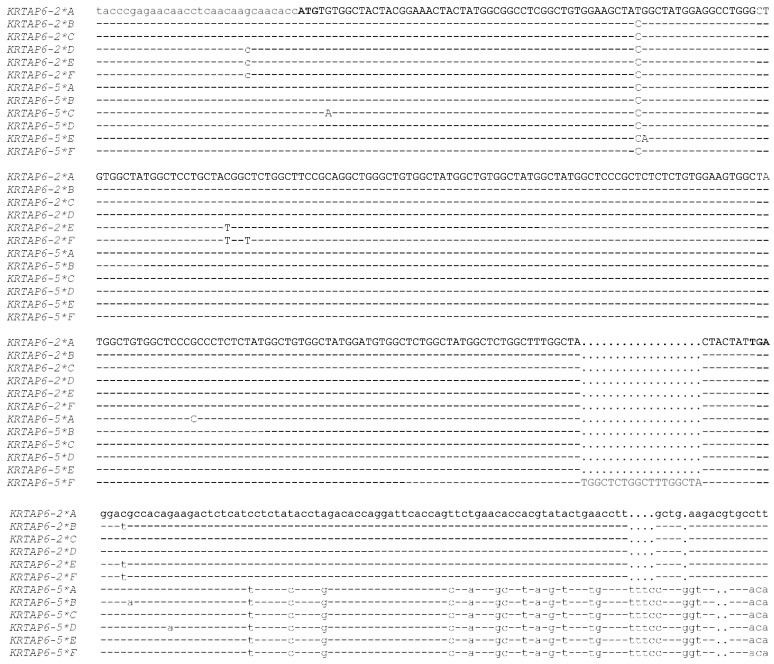
Sequence comparison of variants of ovine *KRTAP6-2* and *KRTAP6-5*. The nucleotides within the coding region are shown in upper-case text, while those in the flanking regions are shown in lower-case text. Dashes indicate nucleotide sequences identical to the top sequence, and dots have been introduced to improve the alignment. The start and stop codons are shown in bold.

Within families, the *KRTAP*s can exhibit a high degree of nucleotide sequence similarity, particularly in their coding regions, and some *KRTAP*s even have identical coding sequences. For example, *KRTAP6-2* variants *B*, *C*, and *D* have coding sequences that are identical to *KRTAP6-5* variants *B* and *D* (Figure 2). These genes and their variants can only be differentiated by variation in their 3′ and 5′ flanking sequences.

The high sequence similarity in coding regions can make it difficult (and sometimes impossible) to assign KAP protein sequences or partial gene sequences into families. Equally, it is also difficult to determine whether different KAP protein sequences represent different family members, or just variant sequences of the same family member. This hampers the application of proteomic approaches to KAP research. It also highlights the critical importance of having extended and comprehensive gene sequences for the *KRTAP*s, along with an idea of their location on chromosomes, if one is to accurately identify and classify both the KAP and the *KRTAP* sequences.

## 3. Variation in the *KRTAP*s

Nucleotide sequence variation has been explored for many of the ovine and caprine *KRTAP*s. To date, all of the ovine and caprine *KRTAP*s that have been investigated are polymorphic, but the extent and nature of the polymorphism varies between the genes. Some *KRTAP*s exhibit a low level of polymorphism, such as ovine and caprine *KRTAP7-1* [34,53] and ovine *KRTAP20-2* [41]. Each of these genes has only two sequence variants. On the other hand, some *KRTAP*s possess a high level of polymorphism, such as ovine *KRTAP1-2* [20,21], ovine *KRTAP1-4* [23], and caprine *KRTAP13-3* [48,59], for which nine or more sequence variants have been identified. The majority of *KRTAP*s exhibit a moderate level of polymorphism, with the number of sequence variants ranging from three to eight.

**Figure 3:**
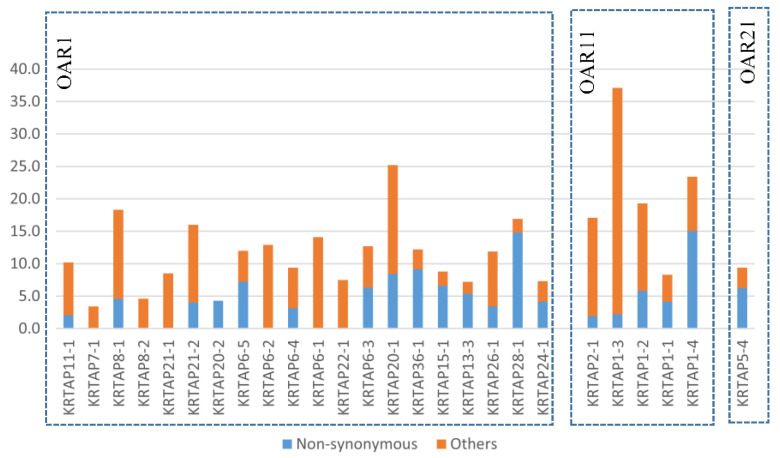
Density of SNPs in selected ovine *KRTAP*s. The density is expressed as the number of SNPs per kilobase. The order of *KRTAP*s on the x-axis represents their relative location on the chromosomes 1, 11, and 21. The SNPs are divided into non-synonymous SNPs (in blue) and others (including synonymous SNPs in the coding region and SNPs in the flanking regions).

The polymorphism described in the *KRTAP*s includes single nucleotide polymorphisms (SNPs), and insertions and deletions (indels). With the exception of *KRTAP6-1*, for which all of the SNPs are found either upstream or downstream of the coding region [31,67], SNPs in all of the other *KRTAP*s mostly occur in the coding region. The SNP density, and the proportion of non-synonymous SNPs, varies considerably between the *KRTAP*s. Some, such as ovine *KRTAP1-3*, *KRTAP1-4*, and *KRTAP20-1*, have a density of over 20 SNPs per kb, while others, such as ovine *KRTAP7-1*, *KRTAP8-2*, and *KRTAP20-2*, have a density of less than five SNPs per kb (Figure 3). Overall, the SNP density in the majority of *KRTAP*s is higher than the average density of 4.9 SNP per kb that has been suggested to occur across the sheep genome [68], albeit that estimate is now quite dated. There does not appear to be any obvious pattern with respect to the chromosomal location of the polymorphism, and this suggests that the generation and accumulation of the SNPs in any given *KRTAP* may be, at least in part, independent of other *KRTAP*s.

The ratio of non-synonymous SNPs to synonymous SNPs does not have any obvious pattern, but the *KRTAP*s that are located close together on the chromosomes appear to have a similar ratio. For example, ovine *KRTAP36-1*, *KRTAP15-1*, and *KRTAP13-3* are located close together on ovine chromosome 1, and they all have a high proportion of non-synonymous SNPs. The same is true for ovine *KRTAP28-1* and *KRTAP24-1* (Figure 3). A low proportion of non-synonymous SNPs is observed for ovine *KRTAP6-2*, *KRTAP6-4*, *KRTAP6-1*, and *KRTAP22-1*, which are clustered in proximity to each other on ovine chromosome 1, and also for ovine *KRTAP1-2* and *KRTAP1-3* on chromosome 11. Further investigation of variation in other *KRTAP*s as they are found and characterized may provide more information about this effect. It also is notable that all of the non-synonymous SNPs revealed to date in the ovine *KRTAP*s are missense, with the exception of a single nonsense SNP in ovine *KRTAP20-2* [41].

**Figure 4:**
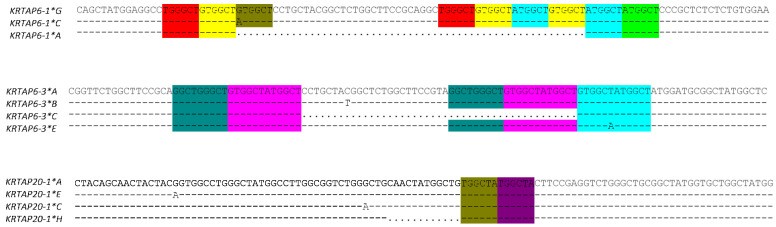
The indels identified in ovine *KRTAP6-1*, *KRTAP6-3*, and *KRTAP20-1*. The sequence repeats flanking the indels are shaded in different colours. In the region that is shown, variants *B*, *D*, *E*, and *F* of ovine *KRTAP6-1* have a sequence identical to ovine *KRTAP6-1* variant *A;* variants *D* and *F* of ovine *KRTAP6-3* have a sequence identical to the ovine *KRTAP6-3* variant *A*; and variants *B*, *D*, *F*, and *G* of ovine *KRTAP20-1* have a sequence identical to ovine *KRTAP20-1* variant *A*; hence, only one variant from the identical sequences is shown.

Beside the presence of SNPs, the *KRTAP*s also contain indels. For sheep, this has been described for numerous *KRTAP*s, including *KRTAP1-1*, *KRTAP5-4*, *KRTAP6-1*, *KRTAP6-5*, *KRTAP20-1*, and *KRTAP28-1* [19,28,29,31,40,47], and *KRTAP9-2* and *KRTAP28-1* in goats [56,66]. For ovine *KRTAP1-1*, *KRTAP5-4*, and *KRTAP6-5*, and for caprine *KRTAP9-2*, the indels occur within tandem repeat regions of the coding sequence, and they lead to variation in the number of tandem repeats that are present. In ovine and caprine *KRTAP28-1*, the indels are located within dinucleotide repeats (microsatellites), while the indels in ovine *KRTAP6-1*, *KRTAP6-3*, and *KRTAP20-1* are not located in the tandem repeat region, but instead occur in regions that are flanked by sequence repeats (Figure 4). All of the indels identified in the *KRTAP*s are in multiples of three nucleotides in size, and hence they preserve the reading frame. The exception is the dinucleotide repeats found in ovine and caprine *KRTAP28-1*.

## 4. Mechanisms for the Generation of *KRTAP* Variation

In the *KRTAP*s, transition SNPs predominate, and they account for over 70% of all SNPs. Among these transition SNPs, the G/C to A/T transition (52.4% of occurrences) happens at nearly three times the frequency of the A/T to G/C transition (18.1%). This effect is still pronounced when the ratio of A/T (approximately 46%) to G/C (approximately 54%) base pairs (1:1.19) in ovine *KRTAP*s is taken into account. Such a transitional bias should create a pressure towards ovine *KRTAP*s having a higher AT content, but some other pressure must operate in the other direction to maintain GC ratio.

For over twenty years, there has been a strong belief that biased gene conversion (BGC) is important for shaping the GC content in the genomes of mammals and other eukaryotes [69]. The BGC theory is based on DNA repair processes inside heteroduplexes, the double-stranded DNA segments that form during meiosis at crossover and non-crossover recombination sites. The theory has it that one DNA strand of a heteroduplex has a maternal origin, while the complementary strand is paternal. The heterozygous sites that occur in heteroduplexes create mismatches, and these mismatches are non-randomly resolved in favour of G/C over A/T nucleotides, which leads to an increase of GC content in the sequence [69,70]. The polymorphic nature of the *KRTAP*s could potentially increase the chance of forming heteroduplexes, and hence elevate BGC. The effect of BGC, if strong enough, can overcome purifying selection and lead to an increased ratio of non-synonymous SNPs to synonymous SNPs [70], as is found for some of *KRTAP*s.

Indels are in abundance in eukaryotic genomes, and in humans they are the second most abundant form of genetic variation, after SNPs [71]. Mechanisms have been proposed to explain the generation of indels, including replication slippage (also known as slipped-strand mispairing) [72], unequal crossing-over (also known as non-reciprocal recombination) [73], and transposition (also known as translocation) [74]. It is thought that replication slippage is the principal mechanism responsible for the majority of small indels in the human genome [75].

Replication slippage is a mutation process that occurs during DNA replication and also during the DNA synthesis step of DNA repair processes. It requires DNA polymerase pausing to occur within a short direct repeat. The paused polymerase dissociates from the DNA, and then the terminal portion of the newly synthesized strand separates from the template and anneals to another direct repeat, after which replication resumes [76]. A slippage event normally occurs when a sequence of repetitive nucleotides (e.g., tandem repeats) are found at the site of replication, and strand misalignment at repeated sequences leads to genetic rearrangements, resulting in the insertion or deletion of nucleotides [77]. Slipped-strand mispairing can also occur with non-continuous repeat sequences, and results in longer insertions or deletions of intervening sequences flanked by the repeats [78].

Many *KRTAP*s are characterized by having an abundance of nucleotide repeats [4,16,19,28,31]. The *KRTAP*s are also GC-rich, with an average GC content of over 54% in all of the ovine *KRTAP*s identified to date. It has been reported that a high GC content results in reduced DNA polymerase processivity and increased DNA polymerase slippage, and consequently it can lead to elevated rates of mutation, including the creation of indels and nucleotide substitutions [79].

There is an association between the occurrence of indels and nucleotide substitutions, and this association appears to be universal to all prokaryotic and eukaryotic genomes examined so far [80,81,82]. McDonald et al. [82] propose that it is not the indels per se, but instead the sequence in which the indels occur that causes the accumulation of nucleotide substitutions. Repeat sequences can promote an increased probability of replication fork arrest and are prone to causing the stalling of high-fidelity DNA polymerase. This can lead to the recruitment of error-prone (low-fidelity) DNA polymerases to replicate the surrounding sequence with a higher-than-average error rate [82].

**Figure 5:**
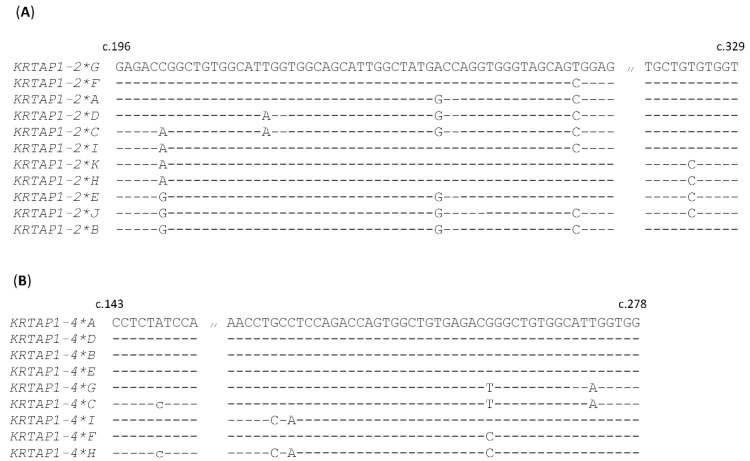
Sequence comparisons revealing potential DNA exchanges between variants of ovine *KRTAP1-2* (**A**) and *KRTAP1-4* (**B**). Nucleotides identical to the top sequences are presented as dashes, and the numbering of the nucleotide positions follows the Human Genome Variation Society (HGVS) nomenclature (http://varnomen.hgvs.org/; accessed on 21 June 2021).

Sequence analyses of two of the most variable *KRTAP*s in sheep (*KRTAP1-2* and *KRTAP1-4*), reveal that short segments of DNA exchange may have occurred and contributed to the generation of the different nucleotide sequences (Figure 5). This suggests gene conversion or non-reciprocal genetic exchange is one of the mechanisms for creating sequence diversity in some *KRTAP*s. Unique sequence motifs have been postulated to promote genetic recombination, including the crossover hotspot instigator (Chi) sequence (5′-GCTGGTGG-3′) or its complementary sequence, which are abundant in the genomes of bacteriophage and *Escherichia coli* [83]. Chi and Chi-like sequences, or their complementary sequences, have been reported in *KRTAP*s [19,61,64,65]. It is proposed that Chi-like sequences, with minor nucleotide variations to the consensus *Escherichia coli* Chi sequence, may have partial ‘hotspot’ activity [84]. The presence of Chi and Chi-like, or their complementary sequences in *KRTAP*s suggests recombination may play a role in creating sequence diversity.

## 5. The Chromosomal Clustering of *KRTAP*s and Evolution

The *KRTAP*s are clustered and located in several chromosomal regions. In humans, where a full complement of *KRTAP*s has possibly been identified, there are five clusters of genes located on three chromosomes [10]. Based on the number of KAP genes in each cluster, ranging from the largest to the smallest, these are: cluster 1, containing 15 KAP gene families (KAP6 to KAP8, KAP11, KAP13, KAP15, and KAP19 to KAP27) located on chromosome 21q22.1; cluster 2, containing seven KAP gene families (KAP1 to KAP4, KAP9, KAP16, and KAP17) located on chromosome 17q21.2; cluster 3, containing two KAP gene families (KAP10 and KAP12) located on chromosome 21q22.3; cluster 4, containing six KAP5 genes (*KRTAP5-1* to *KRTAP5-6*) located on chromosome 11p15.5; and cluster 5, which contains the other six KAP5 genes (*KRTAP5-7* to *KRTAP5-12*) located on chromosome 11q13.4 [10,11].

**Figure 6:**
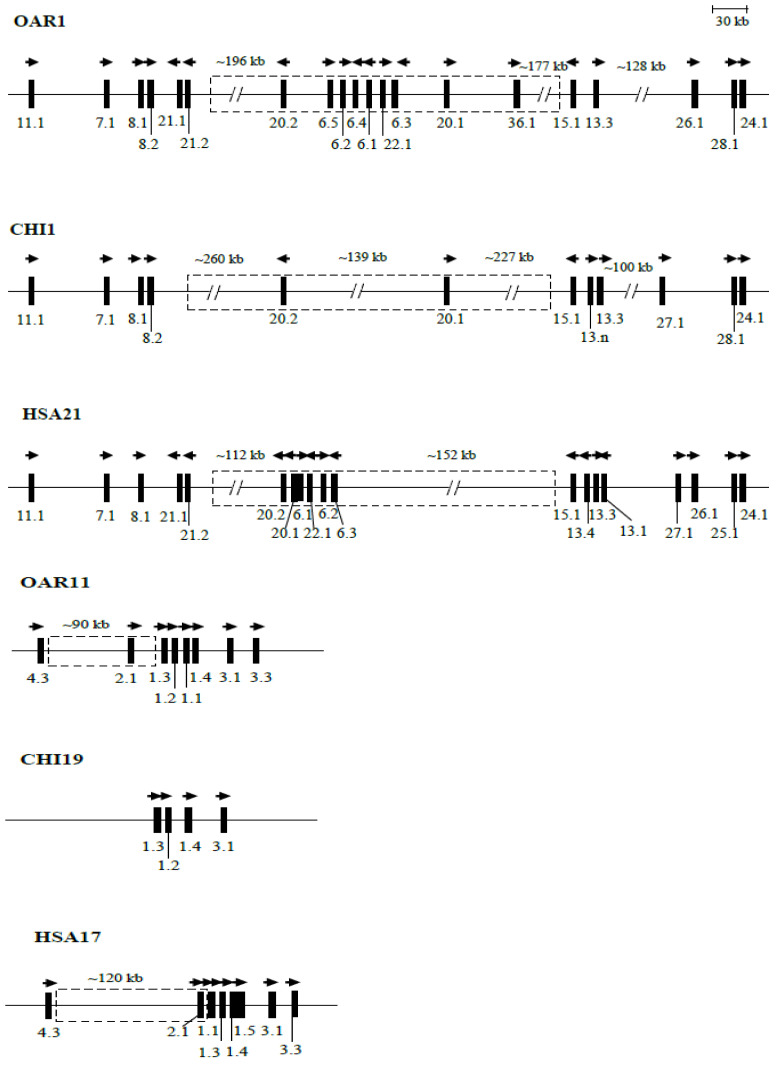
Chromosomal locations of the *KRTAP*s identified in sheep and goats together with their human orthologues. A vertical bar represents each *KRTAP* and the arrowheads above the bars indicate the direction of transcription. The numbers below the bars indicate the name of the *KRTAP*s (i.e., 11.1 represents *KRTAP11-1*). The distances between the *KRTAP*s are only approximate. The dashed-line boxes represent the chromosome regions that appear to be markedly different between sheep/goats and humans. Note that human *KRTAP1-4*, *KRTAP1-3*, *KRTAP1-1*, and *KRTAP1-5* are the orthologues of ovine *KRTAP1-1*, *KRTAP1-2*, *KRTAP1-3*, and *KRTAP1-4*, respectively.

In sheep and goats, all of the *KRTAP*s identified to date are from cluster 1 and cluster 2, except ovine *KRTAP5-4*, a cluster 4 gene on ovine chromosome 21. In these two species, the cluster 1 *KRTAPs* are located on chromosome 1, but the cluster 2 *KRTAP*s are located on chromosome 11 in sheep, and on chromosome 19 in goats (Figure 6).

Within clusters, the *KRTAP*s are unevenly distributed on the chromosome [10,16]. Despite having different transcriptional directions for the *KRTAP*s found in each cluster, the genes that are near to each other tend to have the same direction of transcription (Figure 6 and [5]). This may suggest that some genes are under a shared form of transcriptional control.

The identification of numerous *KRTAP*s from cluster 1 and cluster 2 in sheep and goats enables a comparison of these two ruminant *KRTAP* clusters and the matching human cluster. From this, it can be observed that the overall configuration of *KRTAP*s is similar across these species, but that sheep and goats are more similar to each other (as might be expected), and have some differences to humans. In cluster 1, a major difference is observed in the region between *KRTAP21-2* and *KRTAP15-1*. In humans, this region is estimated to be 306 kb in size, and it contains four KAP gene families (two *KRTAP20*s, three *KRTAP6s*, one *KRTAP22*, and seven *KRTAP19*s; [15]). Genes of the KAP19 family have not been identified in sheep and goats, but for the other *KRTAP*s that have been identified, their locations are quite different to those described in humans (Figure 6). This region is estimated to be 560 kb in length in sheep and contains two additional *KRTAP6*s and one new KAP gene named *KRTAP36-1* [6,31].

An obvious difference in cluster 2 is located between *KRTAP4-3* and *KRTAP1-3* (Figure 6). This region is approximately 120 kb in length in humans, but it is estimated to be 90 kb in size in sheep. At a finer level in humans, *KRTAP2-1* is approximately 5 kb away from human *KRTAP1-1*, which corresponds to ovine *KRTAP1-3*, but the distance between *KRTAP2-1* and *KRTAP1-3* is approximately 28 kb in sheep. This region contains numerous human *KRTAP4*s and *KRTAP2*s [85], but only one *KRTAP4* and one *KRTAP2* have been identified in this region in sheep. The identification of more ovine and caprine *KRTAP*s in this region would assist in providing a better understanding of the similarities and differences between the regions in the different species.

The clustering of genes that produce proteins that are involved in key metabolic pathways has been accepted for many eukaryotes, but the evolutionary causes or benefits of clustering remain controversial [86]. One hypothesis put forward to explain the clustering of genes involved in metabolic pathways is that they have ‘arrived’ in genomes as a group, following horizontal gene transfers from bacteria [87].

Given the intron-less character of the *KRTAP*s, the possibility of individual genes or groups of *KRTAP*s having prokaryotic origins cannot be excluded. Research in humans suggests that the cluster-3 *KRTAP*s are located within introns of the thrombospondin-type laminin G domain and EAR repeat-containing protein gene (*TSPEAR*) on chromosome 21 [88]. This rather strange location could suggest that this *KRTAP* cluster has been inserted into this position, and then subsequently, gene duplication and divergence may have enlarged the cluster.

Gene duplication can arise via several mechanisms, with the major mechanisms including unequal crossing-over, retroposition and chromosomal duplication events (large-scale duplications) [89]. Large-scale duplications, as a consequence of polyploidy, are reported to occur frequently in plants, but are much less frequent in animals [89]. However, duplications of large genomic segments (segmental duplications) are abundant in animals such as primates [90] and rodents [91]. Unequal crossing-over, along with gene conversion, is believed to be the main driver for the generation of gene duplications, but the possibility of retroposition should not be ignored.

Retroposition is an RNA-mediated process that occurs when a message RNA is retro-transcribed to complementary DNA (cDNA), and the resulting cDNA is inserted back into the genome. Retrogenes are therefore expected to lack introns and regulatory sequences (which sits well with the nature of the *KRTAP*s), but instead contain poly A tracts and flanking short direct repeats [89]. Further bioinformatics analyses of the *KRTAP*s and their flanking sequences may shed more light on the evolution of the *KRTAP* clusters.

**Figure 7:**
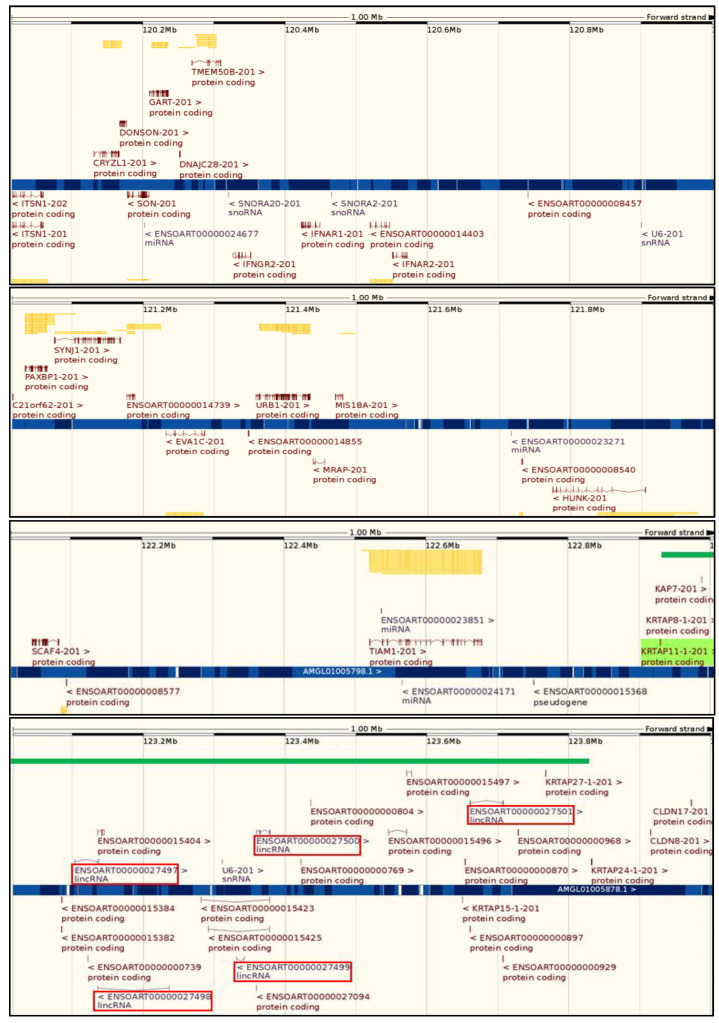

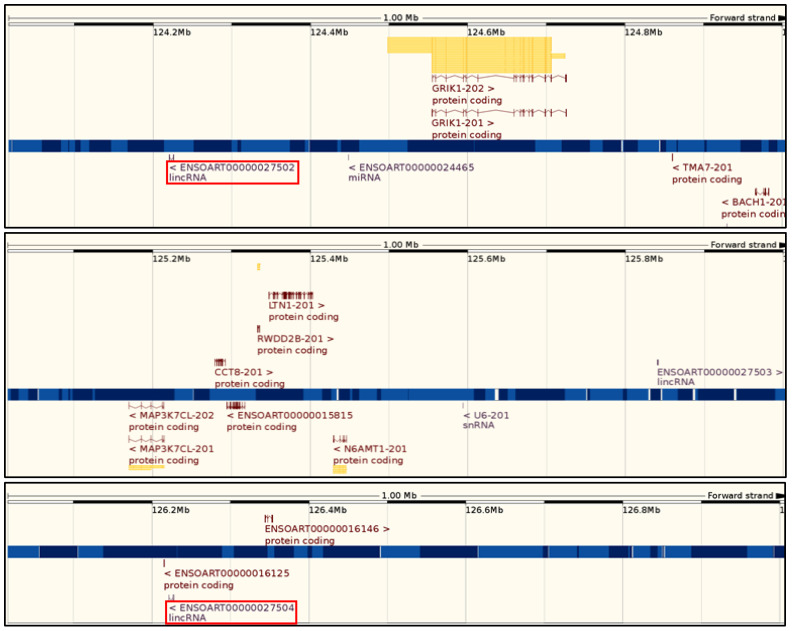
Sequence analysis of the sheep chromosome 1 region reveals the presence of more long intergenic non-coding RNA (lincRNA) genes within the *KRTAP* cluster region than in its flanking regions. The *KRTAP* cluster, spanning from *KRTAP11-1* to *KRTAP24-1*, is indicated by the green bar. The lincRNA genes, identified based on the sheep assembly sequence Oar_rambouillet_v1.0 (GCA_002742125.1) using Ensembl (http://asia.ensembl.org/; accessed on 21 June 2021), are marked in red boxes.

A preliminary sequence analysis of the sheep chromosome 1 region that contains *KRTAP*s reveals five long intergenic non-coding RNA (lincRNA) genes within the cluster region (spanning approximately 0.9 Mb). However, there is no lincRNA gene found in the approximately 2.9 Mb upstream region, and only two lincRNA genes are found in the approximately 3.2 Mb downstream region (Figure 7). The exact functions of lincRNAs are not well known, but it is proposed that they broadly serve to fine-tune the expression of neighbouring genes with tissue specificity, and with a diversity of mechanisms [92]. Analysis of human lincRNAs reveals one feature, a high prevalence of transposable elements (TEs) [93], or repetitive mobile genetic sequences that are capable of duplicating genes or gene fragments [94]. Whether these lincRNAs play a role in the evolution of *KRTAP*s awaits further investigation.

## 6. The Effect of *KRTAP* Variation

The proteins encoded by *KRTAP*s serve as a matrix embedding the KIFs. They form one of the main components of the wool/hair fibre, and thus it is thought that variation in *KRTAP*s may affect fibre properties, possibly in three ways.

Firstly, non-synonymous SNPs and insertions/deletions in the coding region will alter the protein sequence. This may affect the structure and/or properties of the protein, and consequently its interaction with KIFs and/or other KAP proteins, which may then affect fibre properties. As an example, a nonsense mutation in ovine *KRTAP20-2* has been shown to be associated with variation in the curvature of wool fibres [41], and a 57-bp insertion/deletion in the coding region of ovine *KRTAP6-1* is associated with variation in the fibre diameter traits [29]. The mean fibre diameter (MFD) of wool is a key determinant of value, with finer wools of a mean diameter of 19 microns or less being considerably more valuable than strong wools of a mean diameter over 36 microns.

Secondly, synonymous SNPs and SNPs in the upstream and downstream of the coding region may affect gene expression and consequently alter the amount of that protein in the wool/hair fibres. Despite synonymous SNPs not causing amino acid changes in the protein, research has shown that synonymous SNPs can affect the stability and structure of mRNA, and also the folding of protein [95]. In felting lustre mutant wool follicles, Li et al. [96] reported that the expression of *KRTAP2-12* and *KRTAP4-2* was un-regulated, whereas the expression of *KRTAP6-1*, *KRTAP7*, and *KRTAP8* was down-regulated. In wool from sheep on a restricted diet, KAP13-1 and KAP6-n protein levels were increased, and this was found to be associated with a decrease in the fibre diameter [97].

Lastly, variation in *KRTAP*s may also affect the post-translational modification of the protein. A recent proteomic study revealed a differential abundance of some phosphorylated KAPs and keratins, when comparing the crimped and straight wool of Tan sheep [96], and provided evidence that phosphorylation of KAPs and keratins can occur. Bioinformatics analyses of ovine *KRTAP11-1* and *KRTAP13-3* reveals some non-synonymous SNPs that would alter putative phosphorylation sites in proteins derived from these genes [37,38], and the phosphorylation of the KAPs may alter the structural conformation and interactions with KIFs and/or other KAPs, and consequently affect the properties of the wool fibres [98].

## 7. Concluding Remarks and Future Research Directions

To date, 30 *KRTAP*s from 18 different families have been identified in sheep and 18 *KRTAP*s from 12 families have been reported in goats. Most of these genes are present in humans, but some are absent. This suggests that sheep and goats may possess more *KRTAP*s than humans. The ovine and caprine *KRTAP*s are unevenly clustered on chromosomes and translated in alternating directions. The configuration of the *KRTAP*s in the sheep and goat genomes are similar to the configuration reported in humans, but differences occur too. All of the sheep and goat *KRTAP*s are polymorphic, but the extent and nature of polymorphism varies between the genes.

Our current understanding of *KRTAP*s is based primarily on their chromosomal location and sequence, but little is known about how the genes have evolved and the mechanisms that underlie the generation of variation in the genes. Further investigation into their sequences, especially of their flanking regions, may shed more light on their evolutionary origin and how natural selection may have created or enhanced their diversity. It also must not be forgotten that for many hundreds of years humans have been selecting and breeding sheep and goats for fibres, meat, and milk traits; hence, there may evolutionary dead-ends or rare sequences that will be hard, if not impossible, to place in that evolutionary history.

Ongoing investigations are also needed in other important areas. First is the ongoing need to identify and characterize new *KRTAP*s that are likely present in the sheep and goat genomes. This should, in time, lead to the definition of a full catalogue of *KRTAP*s in these species, and the complete annotation of the genes in the reference genomes. This will doubtlessly be enhanced by the use of high-throughput and rapid genome-sequencing techniques, whereby hundreds or thousands of sheep can be rapidly sequenced, subjected to bioinformatics analysis, and publicly recorded and indexed.

Second is the pressing need to better understand the temporal and spatial nature of *KRTAP* expression. Questions about how, when, and why the genes are expressed will need to be addressed, especially if the fibres from sheep and goats are to be improved and better fitted to purpose. Given the potential number of *KRTAP*s that exist, the number of variants for each *KRTAP*, and the diploid nature of the sheep and goat genomes, this will be an immense challenge. If all of the *KRTAP*s are expressed, then there will, by definition, be a large diversity of KAP proteins, and more importantly different permutations of those proteins and the KIFs in the matrix of the fibres. When fully understood, this may enable a greater fibre uniformity to be selected for breeding sheep and goats, and enable us to address one of the bigger constraints on fibre use: its natural variability. This will not be a small task, because while quantitative analytical techniques can be used to investigate whether variation in *KRTAP*s affects gene expression or post-translational modifications of individual *KRTAP*s/KAPs, it is still difficult to unravel the effect of individual *KRTAP*s because of the potentially large numbers of KAP and keratin proteins in fibres. Given the adage, backed by evidence, that ‘there is more variation within any given fleece than between fleeces in any given flock’, the size of this task should not be underestimated. Current studies describing associations between variation in individual *KRTAP*s and variation in fibre traits is a start, but much more research and new multiplex analytical approaches will be needed.

## Figures and Tables

**Table 1:** Summary of the ovine *KRTAP*s that have been identified.

*KRTAP*	Number of Variants	Number of SNPs	Length Variation	GenBank Accession Numbers	References
*KRTAP1-1*	3	3	± 30-bp repeats	L33885-L33887	[19]
*KRTAP1-2*	11	10	No	HQ897973-HQ897982, KM105941-KM105942	[20,21]
*KRTAP1-3*	9	17	No	AY835589-AY835597	[22]
*KRTAP1-4*	9	14	No	GQ507741-GQ507749	[23]
*KRTAP2-1*	4	9	No		[24]
*KRTAP3-1*	Unknown	Unknown	Unknown	M21099	[25]
*KRTAP3-3*	Unknown	Unknown	Unknown	N21103	[25]
*KRTAP4-3*	Unknown	Unknown	Unknown	EU239778	[26]
*KRTAP5-1*	Unknown	Unknown	Unknown	X55294	[27]
*KRTAP5-4*	5	6	±30-bp repeats	GU255997-GU256001	[28]
*KRTAP6-1*	5	4	± 57-bp	GU319873, GU319875	[29,30]
*KRTAP6-2*	6	5	No	KT725827-KT725832	[31]
*KRTAP6-3*	7	5	± 45-bp	KT725833-KT725837, GU319876	[31,32,33]
*KRTAP6-4*	3	3	No	KT725838-KT725840	[31]
*KRTAP6-5*	6	5	± 18-bp	KT725841-KT725846	[31]
*KRTAP7-1*	2	1	No	JN091630, JN091631	[34]
*KRTAP8-1*	5	4	No	JN091632-JN091636	[34]
*KRTAP8-2*	3	2	No	KF220646-KF220647	[35,36]
*KRTAP11-1*	6	5	No	HQ595347-HQ595352	[37]
*KRTAP13-3*	5	4	No	JN377429-JN377433	[38]
*KRTAP15-1*	4	4	No	MH742372-MH742375	[39]
*KRTAP20-1*	8	6	±12-bp	MH243552-MH243559	[40]
*KRTAP20-2*	2	1	No	MH071391, MH071392	[41]
*KRTAP21-1*	3	2	No	MF143980-MF143983	[42]
*KRTAP21-2*	5	4	No	MF143975-MF143979	[43]
*KRTAP22-1*	3	2	No	KX377616-KX377618	[44]
*KRTAP24-1*	4	7	No	JX112014-JX112017	[45]
*KRTAP26-1*	4	7	No	KX644903–KX644906	[46]
*KRTAP28-1*	6	8	±2-bp repeats	MN053915-MN053920	[47]
*KRTAP36-1*	3	4	No	MK770620-MK770622	[6]

**Table 2:** The caprine *KRTAP*s that have been identified.

*KRTAP*	Number of Variants	Number of SNPs	Length Variation	GenBank Accession Numbers	References
KRTAP1-1 *	7	5	No		[48]
*KRTAP1-2*	6	5	±60-bp and 15-bp		[49]
KRTAP1-3 *	Unknown	Unknown	Unknown	JQ772533	[50]
*KRTAP1-4*	6	8	No	N012101, JN012102, JN000317, JN000318, JQ436929, JQ627657	[51]
*KRTAP3-1*	Unknown	Unknown	Unknown	NM_001285774	[52]
*KRTAP7-1*	2	1	No	AY510121	[53]
*KRTAP8-1*	4	2	No	AY510122, EU595394, EU595395	[48,54]
*KRTAP8-2*	3	2	No	AY510123	[55]
*KRTAP9-2*	3	1	±30-bp	AY510124, EU430080,	[56,57]
*KRTAP11-1*	Unknown	Unknown	Unknown	JQ795995	[58]
*KRTAP13-3*	18	17	No	JX426138-JX426145	[48,59]
*KRTAP13-n*	2	1	No	AY510115	[60]
*KRTAP15-1*	6	8	No		[61]
*KRTAP20-1*	4	6	No	MG742218- MG742221	[62]
*KRTAP20-2*	3	4	No	MF973462-MF973464	[63]
*KRTAP24-1*	4	9	No	MG996011-MG996014	[64]
*KRTAP27-1*	3	2	No	MN934937- MN934939	[65]
*KRTAP28-1*	5	8	±2-bp repeats		[66]

* The gene appears to have been identified, but is not well characterised.

## Data Availability

Not applicable.
